# A molecular renaissance in Alzheimer's disease research: The rise of systems biology and spatial omics

**DOI:** 10.1177/13872877251387169

**Published:** 2025-10-17

**Authors:** L Monserrat Perez-Navarro, Juan Carlos Lopez-Alvarenga

**Affiliations:** 1 Servicio de Nefrología, Hospital General de México Dr Eduardo Liceaga, Mexico City, Mexico; 2 Division of Population Health & Biostatistics, School of Medicine, University of Texas Rio Grande Valley, Edinburg, TX, USA

**Keywords:** Alzheimer's disease, bioinformatics, machine learning, omics, transcriptomics

## Abstract

Recent advances in multi-omics and spatial proteomics are reshaping our understanding of Alzheimer's disease. Guo et al.^1^ applied integrative multi-omics to stratify mild cognitive impairment into biologically distinct subtypes with divergent progression trajectories: one metabolically impaired and slow-progressing, the other immune-activated and rapidly declining. Current techniques such as STC-DESI, LCM-MS, and machine learning enhance regional proteomic resolution, supporting biomarker discovery and spatially targeted interventions. This work exemplifies a broader shift toward precision medicine and a systems-level molecular framework in neurodegenerative disease research.

We are living through a new renaissance in biomedical science. Just as Renaissance thinkers merged art, anatomy, engineering, and philosophy to reshape our understanding of humanity, today's scientists are integrating diverse biological “languages” like transcriptomics, epigenomics, and metabolomics, among other -omics. Today, these biological approaches are being aggregated to reinterpret complex diseases such as Alzheimer's disease and related dementias (ADRD). The recent study by Guo et al.,^1^ characterizes this convergence, offering a unified molecular view of AD progression.

In their publication, Guo et al.^1^ applied a multi-omics integration strategy to stratify individuals with mild cognitive impairment (MCI) into two distinct biological subtypes. One predicted subtype is characterized by metabolic dysregulation and slower progression to AD, while the other exhibits chronic immune activation and more rapid decline. This dual-pathway model captures the underlying heterogeneity of AD and highlights the need for biologically informed patient stratification in both clinical practice and research.

Differentiating this heterogeneity has clinical implications, such as enabling more aggressive monitoring of individuals with Subtype 2, who are at higher risk for rapid deterioration, and guiding more effective interventions. Importantly, these findings align with a growing body of literature in which omics integration provides unprecedented insight into AD pathophysiology.

For example, multi-omics studies analyzing cerebrospinal fluid (CFS)^2–4^ and saliva^5,6^ are helping to identify biomarkers with diagnostic and prognostic potential. Similarly, machine learning approaches that combine omics with neuroimaging have revealed complex structural and metabolic patterns, including biomarkers and brain volume indicators, which are indicative of disease progression.^7^

Recent technological advances, such as spatially resolved multi-omics using segmented temperature-controlled or spatiotemporal controlled DESI (STC-DESI) or laser capture microdissection and mass spectrometry (LCM-MS), are enabling region-specific profiling of brain proteomes, including areas affected by amyloid plaques and tau tangles^8^ (Figure 1). These techniques not only enhance our understanding of regional vulnerability in AD but also inform the design of localized therapeutic strategies.

**Figure 1.**
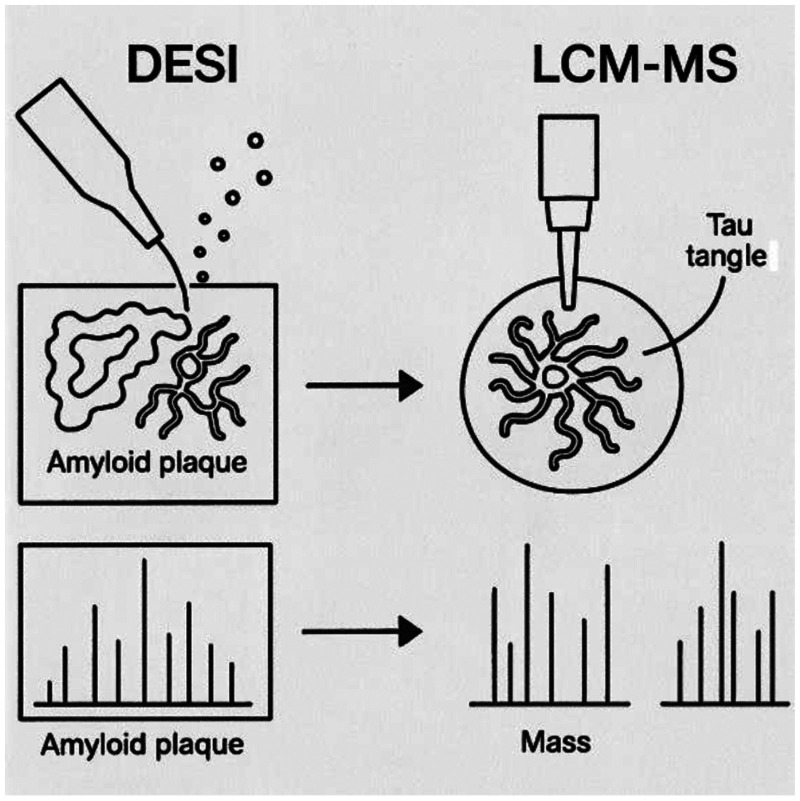
The left side shows a DESI probe spraying charged droplets on a tissue, interacting with molecules on the surface. The ions are interpreted in a mass spectrometer, reflecting the molecular composition. On the right side, a laser microdissection technique is used to isolate a specific region, which is then processed for mass spectrometry.

These advanced techniques, when combined with artificial intelligence (AI), allow high-resolution mapping (or cartography) of brain proteomics at the cellular and regional levels. The high-dimensional dataset obtained through machine learning analysis can reveal molecular signatures correlated with disease progression, cognitive decline, or treatment. This integrative approach would deepen our understanding of AD physiopathology and open the gates for new biomarkers and spatially targeted therapies.

While conceptual distinctions between fast and slow decliners have been proposed in previous research, this study is among the first to define such trajectories using robust multi-omic integration. This approach enabled the identification of immune microenvironments, regulatory gene networks, and prognostic biomarkers that are unique to each subtype. The performance of their subtype-specific progression models is also noteworthy, achieving AUROC values ranging from 0.85 to 0.93 for progression models, which are high. However, the curves do not indicate specific diagnosis cutoff points; therefore, external validation is necessary, as the trajectory subtypes have been conceptually explored before (e.g., slow versus fast decliners), but not as clearly defined molecularly using integrated omics. Importantly, the reproducibility of the study in other populations or with different environmental factors can result in varying results across cohorts.

The authors highlighted their plasma scalable approach but missed early studies on CSF omics that showed heterogeneity in AD due to molecular alterations in proteomic and metabolic pathways linked to amyloid and tau pathology.^9^ These efforts demonstrate the opportunity for analyzing preclinical detection of disease process.^10^ Large-scale plasma omics complements such studies at the population level by enabling the scalable development of biomarkers. Together, CSF and plasma omics complement the translational application of biological frameworks in AD in primary care.^11^

In conclusion, Guo et al.^1^ study reflects a broader shift in AD research toward systems-level biology and precision medicine. By integrating multiple molecular dimensions, we are beginning to decode the biological heterogeneity of AD in ways that may soon translate into real-world diagnostic and therapeutic tools. In this sense, these multi-omics approaches are not only scientifically significant but also emblematic of a molecular renaissance in our understanding of neurodegenerative disease.
